# CSPP-L and EB3 localize to centriolar satellites and are required for satellite-dependent recruitment of ciliopathy proteins to the centrosome

**DOI:** 10.1186/2046-2530-4-S1-P76

**Published:** 2015-07-13

**Authors:** J Sternemalm, S Geimer, EK Aarnes, KM Frikstad, T Stokke, LB Pedersen, S Patzke

**Affiliations:** 1 Institute for Cancer Research, Dept Radiation Biology, OUH - Norwegian Radium Hospital, Oslo, Norway; 2 Cell Biology / Electron Microscopy, University of Bayreuth, Bayreuth, Germany; 3 Department of Biology, University of Copenhagen, Copenhagen, Denmark

## Objective

*Centrosome/Spindle Pole associated Protein 1 *(*CSPP1, JBTS21*) mutations cause Joubert syndrome (JBTS) and JBTS-related ciliopathies. The large protein isoform CSPP-L is a ciliary protein required for ciliogenesis and stabilization of the ciliopathy protein RPGRIP1L (NPHP8/JBTS7/MKS5/FTM) at the ciliary transition zone (TZ). However, RPGRIP1L is dispensable for ciliogenesis and the mechanism by which CSPP-L promotes ciliogenesis is unclear.

## Methods

We applied immunogold electron, immunofluorescence and fluorescence live cell microscopy to determine localization of CSPP-L at high spatial and temporal resolution. We elucidated the functional interplay of CSPP-L with centriolar satellites in hTERT-RPE1 and HeLa cells using biochemical analysis of CSPP-L complexes, siRNA modulated gene expression and quantitative immunofluoresecence microscopy.

## Results

We show that CSPP-L localizes to centriolar satellites, in addition to axonemal microtubule (MT) plus ends and the TZ, and that the MT plus end-tracking protein EB3 also localizes to satellites. CSPP-L complexed with the known satellite component PCM1 and GFP-CSPP-L showed satellite-like dynamics. Importantly, CSPP-L depletion decreased formation of PCM1, CEP290 and EB3-comprising satellites, whereas depletion or inactivation of EB3 impaired centrosomal localization of CSPP-L.

## Conclusion

Our results identify a new link between MT plus ends and centriolar satellites, and suggest that CSPP-L contributes to ciliogenesis by promoting EB3- and dynein-dependent recruitment of satellite components to the centrosome.

